# Comparing HemoCue® and Quantitative Buffy Coat® and Coulter Counter-measured haemoglobin concentrations in African children with acute uncomplicated malaria: a Bland–Altman analysis

**DOI:** 10.1186/s12936-025-05318-5

**Published:** 2025-03-11

**Authors:** Dhol S. Ayuen, Peter Olupot-Olupot, Rita Muhindo, Marie A. Onyamboko, Seun Ajayi, Natenapa Chimjinda, Chiraporn Taya, Sophie Uyoga, Thomas N. Williams, Kathryn Maitland, Caterina Fanello, Nicholas P. J. Day, Walter R. Taylor, Mavuto Mukaka

**Affiliations:** 1 Centre for Tropical Medicine and Global Health, Nuffield Department of Medicine, University of Oxford, Oxford, UK; 2 Mahidol Oxford Tropical Medicine Research Unit (MORU), Faculty of Tropical Medicine, Mahidol University, 420/6 Rajvithi Road, Bangkok, 10400 Thailand; 3 Mbale Clinical Research Institute (MCRI), P.O. Box 1966, Mbale, Uganda; 4 Busitema University, P.O. Box 1460, Mbale, Uganda; 5 Kinshasa School of Public Health, University of Kinshasa, Avenue Tombalbaye 68-78, Kinshasa, Democratic Republic of Congo; 6 KEMRI-Wellcome Trust Research Programme, Kilifi, Kenya; 7 Institute of Global Health Innovation, Department of Surgery and Cancer, Imperial College London, London, SW7 2AS UK

**Keywords:** Bland–Altman analysis, Haemoglobin, Anaemia, Malaria, HemoCue, Coulter Counter, QBC®

## Abstract

**Background:**

Anaemia is a deleterious consequence of malaria, and its accurate diagnosis is crucial for effective management. However, laboratory methods for measuring haemoglobin (Hb) concentration, like the Coulter Counter and the Quantitative Buffy Coat® (QBC®), are costly and not widely accessible in resource-limited settings. The point-of-care HemoCue® test is a cheaper alternative and suitable in rural areas. The study aimed to determine the level of agreement between Coulter Counter/QBC® vs. HemoCue®-measured Hb concentrations by Bland–Altman analysis.

**Methods:**

As part of a randomized, placebo-controlled trial of single low-dose primaquine in Ugandan and Congolese children with acute uncomplicated *Plasmodium falciparum* malaria, Hb concentrations were measured on days 0, 3, 7, and 28 using Coulter Counter (Uganda, n = 1880 paired values), QBC® (DR Congo, n = 1984 paired values) and HemoCue® Hb-301™. The predefined clinically acceptable limits were set at ± 0.5 g/dL.

**Results:**

The Bland–Altman analysis showed that the HemoCue® minus Coulter Counter mean Hb difference was − 0.15 g/dL with lower and upper limits of agreement of − 3.68 g/dL and 3.39 g/dL, respectively. Corresponding HemoCue® minus QBC® values were − 0.23 g/dL, − 1.66 g/dL and 1.22 g/dL. Linear regression of Hb concentration differences vs. mean Hb concentrations showed negative correlations: r = − 0.43 and r = − 0.34 for HemoCue® vs. Coulter Counter and HemoCue® vs. QBC®, respectively.

**Conclusions:**

Compared to Coulter and QBC®, mean HemoCue® measured Hb concentrations were lower and, compared to the Coulter or QBC® methods, had an overall tendency to measure lower Hb concentrations with increasing Hb concentrations. Upper and lower limits of agreement were wider than the predefined clinically acceptable limits of ± 0.5 g/dL. HemoCue® should be used with caution in settings where decisions about blood transfusions are made.

**Supplementary Information:**

The online version contains supplementary material available at 10.1186/s12936-025-05318-5.

## Background

Malaria remains a significant global public health threat. In 2022, 249 million malaria cases were reported, increasing from the previous year’s 247 million [1]. The majority, 94%, of cases were concentrated in the WHO African Region, with four countries—Nigeria, the Democratic Republic of Congo (DRC), Uganda, and Mozambique—accounting for nearly half of all cases. *Plasmodium falciparum* is the predominant species globally and accounted for most of the 608,000 malaria-related deaths in 2022.

Anaemia is a particular health challenge in sub-Saharan Africa [2], where malaria, poor feeding practices, inherited blood disorders, and micronutrient deficiencies contribute to the disproportionate anaemia rates in infants, young children, and pregnant women [3, 4]. The prevalence of anaemia among children under five years in Uganda is 58.8% [5] and 68% in the DRC [6]. Malarial-related anaemia involves several mechanisms that include intravascular haemolysis of parasitized red blood cells, the splenic removal of non-parasitized red blood cells [7–10], dyserythropoiesis (abnormal red blood cell production), bone marrow suppression [11, 12], co-infections with bacteria, HIV-1, and hookworm [13, 14], and the cumulative effect of multiple malaria infections in high-endemic areas [4].

Severe malarial anaemia (SMA) in African children < 12 years due to *P. falciparum* is defined as a haemoglobin (Hb) concentration < 5.0 g/dL or haematocrit < 15.0% in the presence of a parasite density > 10,000/µL [15] and contributes significantly to inpatient fatalities in referral centres and the need for blood transfusions, notably in young children 2 to 12 years [16–18]. The accurate diagnosis of anaemia is crucial for effective management and only the measured Hb concentration can classify patients with mild, moderate, and severe anaemia [19]. Conjunctival, palmar, and nailbed pallor are subjective and insensitive for diagnosing mild or moderate anaemia [20, 21].

Several methods exist for measuring Hb, including the cyanmethaemoglobin method (the reference method), gasometric techniques, specific gravity methods, chemical assays, Coulter Counter and Quantitative Buffer Coat® (QBC®). However, the Coulter Counter is the most widely available method and is used commonly in many laboratories [22]. It also has the advantage of measuring crucial blood indices, white blood cells and platelets. The QBC® is an alternative to the Coulter Counter and is a valuable and rapid method for measuring the Hb. It is also accurate and reliable. In the QBC® method, a blood sample is mixed with a specialized stain and centrifuged in a QBC® tube; the fluorescence intensity of the erythrocyte layer determines the Hb concentration. Despite their accuracy, these methods are costly, time-consuming and require technical expertise, hence, they are not readily available in most resource-limited settings, especially in small clinics and health posts. This makes diagnosis and management of anaemia challenging in these settings.

Portable photometric point-of-care analysers, such as HemoCue®, have been developed to measure Hb concentrations using small-volume blood samples. These devices are cheap, user-friendly and offer rapid results, making them suitable for diverse healthcare settings [23, 24]. However, the accuracy and reliability of HemoCue® is uncertain. To use interchangeably HemoCue® with the Coulter Counter and QBC® methods in resource-limited settings, its accuracy should approximate the accuracies of the Coulter Counter or QBC®.

Previous studies have shown varying levels of agreement between HemoCue®- and Coulter Counter-measured Hb concentrations, with a mix of acceptable and unacceptable levels of agreement that have been determined by individual researchers. Most studies suffer from small sample sizes and consequential limited power, leading to inconclusive Bland–Altman plots [25–31]. Nevertheless, these studies leave open the question of the accuracy and reliability of HemoCue® in resource-limited settings. Moreover, no study, to the best of available evidence, has compared HemoCue® against QBC®. This adequately sized study, therefore, sets out to determine the accuracy of HemoCue® Hb-301™ vs. Coulter Counter and vs. QBC in two sub-Saharan African countries.

## Methods

### Study design, participants and site

This is a secondary analysis of data from a randomised, placebo-controlled trial of single low-dose primaquine in combination with either artemether-lumefantrine or dihydroartemisinin-piperaquine, conducted at the Mbale Regional Referral Hospital (MRRH), eastern Uganda, and the Kinshasa Mahidol Oxford Research Unit (KIMORU) in DRC [32]. Children were enrolled if they were aged between 6 months and 11 years, presented with clinically uncomplicated disease, with a measured fever (≥ 37·5 °C aural) or a history of fever ≤ 72 h and Hb concentration ≥ 6 g/dL, and either a positive malaria slide for *P falciparum* (mono or mixed infection) or, in Uganda only, a positive rapid diagnostic test (SD Bioline Malaria Ag Pf/Pan test, SD BioLine, Suwon, South Korea).

### Ethical approvals

Ethical approvals were obtained from the following ethics committees: the Oxford University Tropical Research Ethics Committee (reference 53-16), the Mbale Regional Referral Hospital Institutional Review Committee (MRRH-REC OUT—COM 006/2017), the Uganda National Drug Authority (CTA00280), and the Uganda National Council for Science and Technology (HS2205). In the DRC, approvals were granted by the Ministry of Higher and University Education, the University of Kinshasa Public Health School Ethics Committee, and the City of Kinshasa Provincial Government Health Minister (ref 135/MIN.SAN.AFFSOC&ACHUM/CM/JD/2017).

### Study procedures

Hb concentrations on days (D) 0, 3, 7 and 28 were measured by finger prick capillary samples for the HemoCue 301® Hb-301™ (HemoCue®, Angelholm, Sweden) and venous blood samples for a full blood count using the Coulter Counter in Uganda (DxH500, Beckman Coulter, Indianapolis, IN, USA) and the QBC® method in KIMORU.

### Data collection and statistical analysis

Data were collected on standardized case report forms and entered into MACRO, a web-based clinical data management system. Data analysis was performed in R (Version R 4.3.1). Descriptive statistics were used to summarize participant baseline characteristics.

Agreements between HemoCue® and Coulter Counter/QBC® measurements were assessed using the Bland–Altman method by: (i) plotting a graph of the Hb differences between paired Hb results of HemoCue® minus Coulter and HemoCue® minus QBC® (y axis) against the mean Hb concentration of the two paired methods, and (ii) calculating the: (a) overall mean Hb difference between the paired methods, (b) upper and lower limits of agreement, (c) standard deviations (SD) and standard errors (SE) and 95% confidence intervals (CIs) around the mean difference and limits of agreement.

Clinically acceptable limits of agreement were set a priori at ± 0.5 g/dL. These tight limits were chosen because, clinically, a greater emphasis was placed on not missing a diagnosis of anaemia.

Pearson correlation coefficients were also calculated to evaluate the relationship between differences in paired Hb concentrations and mean Hb concentrations, using scatter plots for visual inspection. Histograms and Quantile–Quantile plots were used to inspect the distribution of Hb differences between the two measurements and baseline Hb measurements to assess for normality.

## Results

A total of 966 participants had paired Hb measurements from Mbale and Kinshasa (Table 1); their baseline characteristics were similar across both sites. Overall, there were 1880 pairs of Hb concentrations from Mbale and 1984 from Kinshasa across the four time points; they were all approximately normally distributed, irrespective of the Hb measurement method used (Additional file 1. Supplementary Figures for the Bland–Altman Analysis: Fig. S1 and Fig. S2). The mean D0 HemoCue® Hb was 10.8 g/dL (range: 6.2 g/dL to 15.1 g/dL) in Mbale and 10.4 g/dL (range: 6.0 g/dL to 14.0 g/dL) in Kinshasa; subsequent concentrations are shown in Table 2.

**Table 1:** Participant baseline characteristics

Study arm	Overall	Mbale, Uganda	Kinshasa, DRC
Number of participants (N)	966	470	496
Age (years) (median (IQR))	5.0 (3.0,8.0)	5.0 (2.0,8.0)	5.0 (3.0,7.0)
*P. falciparum* parasitaemia (per μl) (median (IQR))	36,989 (2085;118,044)	36,958 (2164;112,663)	36,989 (1894;125,114)
Sex:			
Female (%)	399/966 (41.3)	197/470 (41.9)	202/496 (40.7)
Male (%)	567/966 (58.7)	273/470 (58.1)	294/496 (59.3)
Weight (kg) (median (IQR))	17.0 (12.7;22.1)	17.0(12.5;22.0)	17.0 (12.8;23.0)
Splenomegaly:			
Present (%)	226/966 (23.4)	106/470 (22.6)	120/496 (24.2)
HemoCue® haemoglobin (g/dl) (mean (SD))	10.6 (1.6)	10.8 (1.7)	10.4 (1.5)
Coulter Counter & QBC® haemoglobin (g/dL) (mean (SD))	10.7 (2.1)	10.9 (2.5)	10.7 (1.7)
Genotypic G6PD status:			
Not done (%)	13/966 (1.3)	4/470 (0.9)	9/496 (1.9)
Deficient (%)	241/966 (25.0)	110/470 (23.4)	131/496 (26.4)
Normal (%)	609/966 (63.0)	304/470 (64.7)	305/496 (61.5)
Trait (%)	102/966 (10.6)	52/470 (11.1)	50/496 (10.1)
Undetermined (%)	1/966 (0.1)	0/470 (0.0)	1/496 (0.2)
Genotypic sickle cell status:			
Positive AS (sickle cell trait) (%)	140/966 (14.5)	74/470 (15.7)	66/496 (13.3)
Positive SS (sickle cell disease) (%)	2/966 (0.2)	0/470 (0.0)	2/496 (0.4)
Genotypic alpha-thalassemia status:			
Normal (not thalassaemic) (%)	460/966 (47.6)	230/470 (48.9)	230/496 (46.4)
Heterozygous (single deletion) (%)	412/966 (42.7)	193/470 (41.1)	219/496 (44.2)
Homozygous (double deletion) (%)	94/966 (9.7)	47/470 (10.0)	47/496 (9.5)

**Table 2:** Means of HemoCue®-measured and Coulter Counter-and QBC-measured haemoglobin concentrations on different days in Mbale and Kinshasa

Mbale–Uganda days	HemoCue®	Coulter Counter
	n = 470	n = 470
Day 0 mean (SD)	10.8 (1.7)	10.9 (2.5)
Day 3 mean (SD)	10.0 (1.6)	10.2 (2.3)
Day 7 mean (SD)	10.4 (1.4)	10.6 (2.2)
Day 28 mean (SD)	11.5 (1.3)	11.8 (1.9)
All days combined	10.7 (1.6)	10.8 (2.3)

Kinshasa—DRC days	HemoCue®	QBC®
	n = 496	n = 496
Day 0 mean (SD)	10.4 (1.5)	10.7 (1.7)
Day 3 mean (SD)	9.7 (1.5)	10.0 (1.7)
Day 7 mean (SD)	10.1 (1.3)	10.3 (1.5)
Day 28 mean (SD)	11.7 (0.9)	12.1 (1.1)
All days combined	10.5 (1.5)	10.7 (1.7)

The range of HemoCue®-measured Hb concentrations for all days in Mbale was 5.5 g/dL to 16.2 g/dL and 5.3 g/dL to 14.2 g/dL in Kinshasa, and 1.7 g/dL to 24.0 g/dL (Coulter) and 5.0 g/dL to 15.6 g/dL (QBC®).

Scatterplots of HemoCue®- vs. Coulter-measured and HemoCue®- vs. QBC®-measured Hb concentrations showed positive correlation coefficients of 0.63 (p < 0.0001, Mbale) and 0.91 (p < 0.0001, Kinshasa) (Additional file 1. Supplementary Figures for the Bland–Altman Analysis: Fig. S3).

### The Bland–Altman analysis

**Fig. 1:**
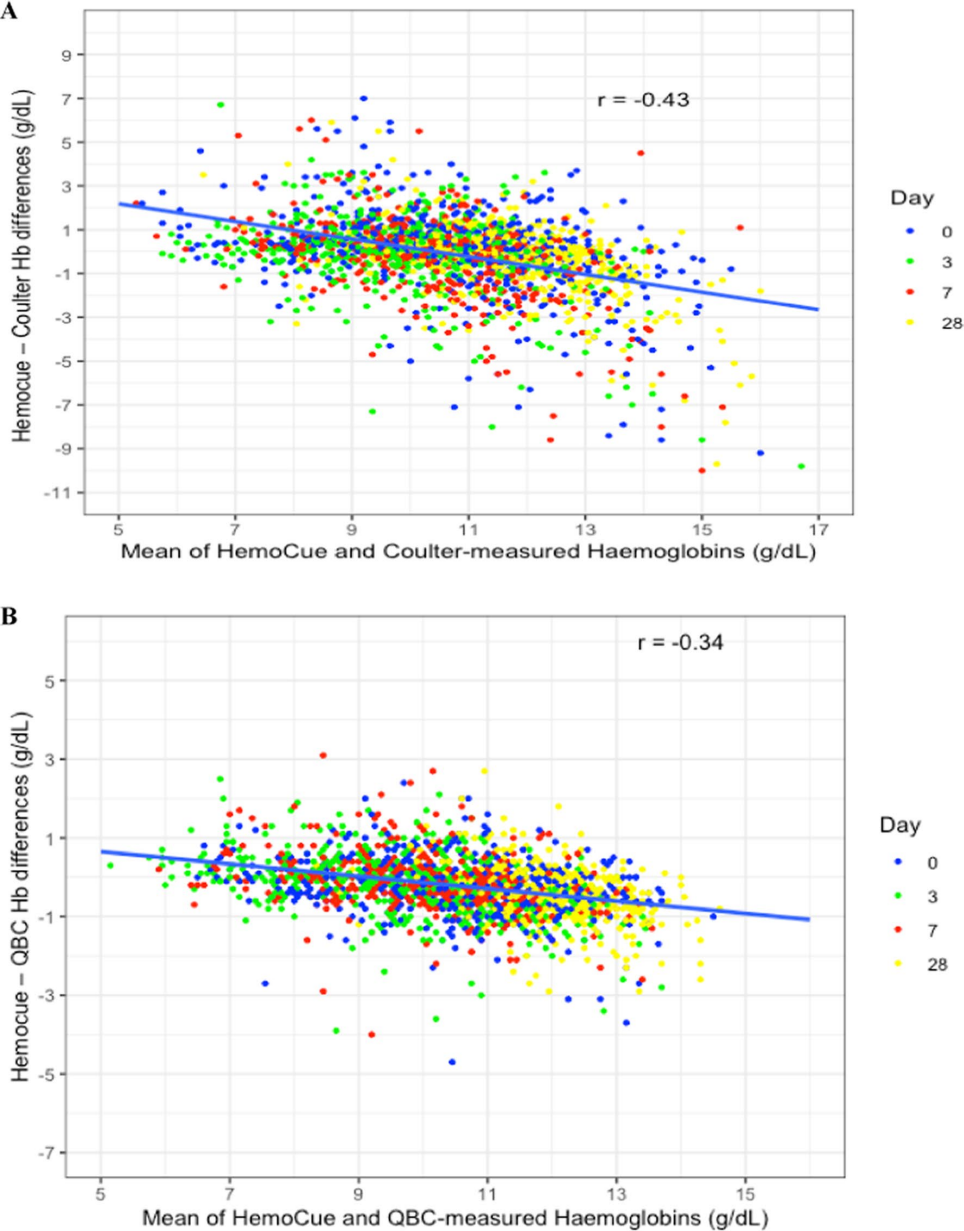
Scatter plots of the differences of HemoCue®—Coulter Counter (**A**) and HemoCue®—QBC® (**B**) and means of HemoCue® and Coulter Counter/QBC®-measured haemoglobin concentrations

The scatterplots of Hb differences against mean Hb concentrations of HemoCue® vs. Coulter and HemoCue® vs. QBC® showed negative correlation coefficients of − 0.43 (p < 0.0001, Mbale) and − 0.34 (p < 0.0001, Kinshasa) (Fig. 1). The differences in Hb concentrations (HemoCue® vs. Coulter/QBC®) were also approximately normally distributed (Additional file 1. Supplementary Figures for the Bland–Altman Analysis: Figs. S4 and S5). Therefore, the assumptions of the Bland–Altman analysis were met, and the analysis proceeded without log-transforming the data.

### Mbale, Uganda

**Fig. 2:**
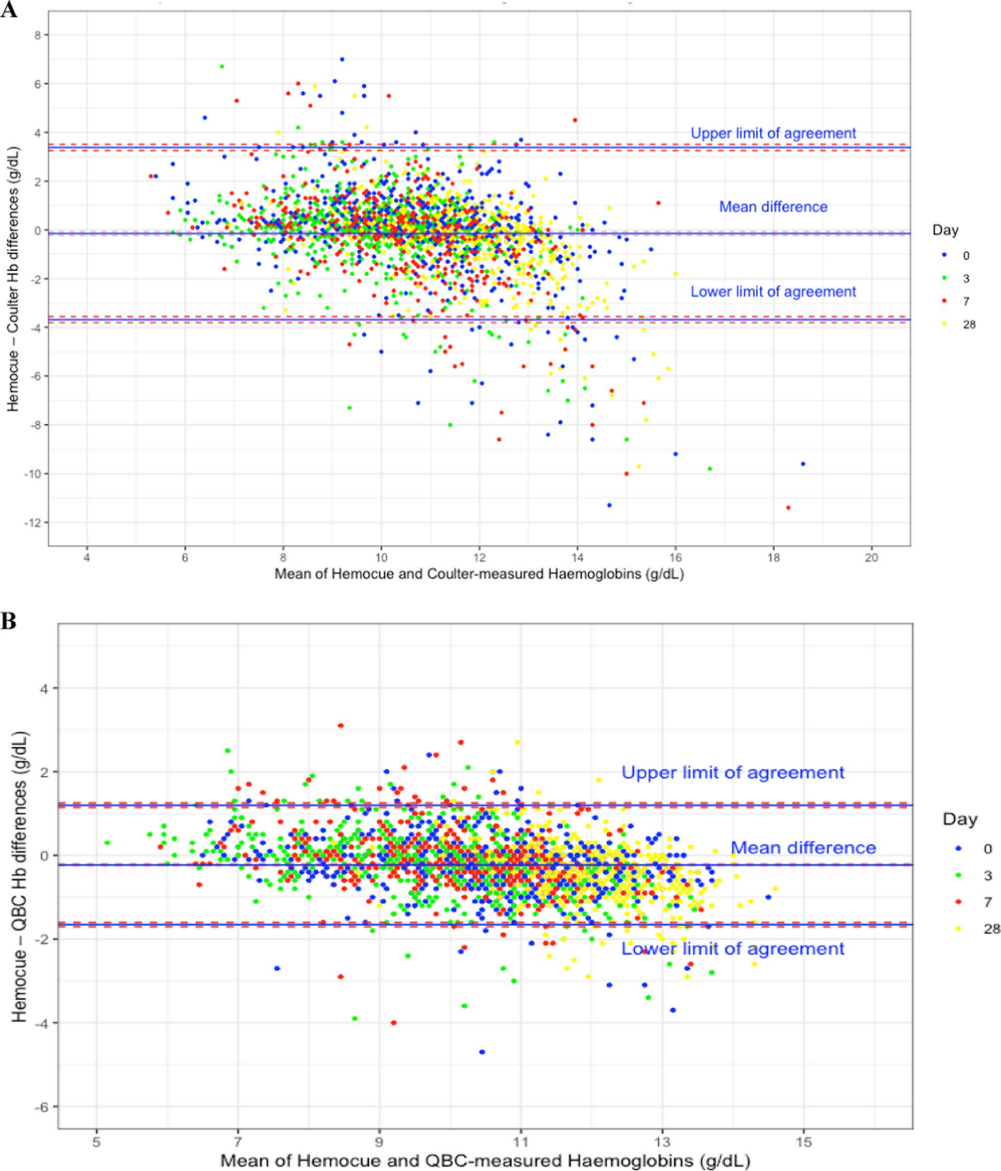
Bland–Altman plots* of HemoCue®—Coulter Counter (**A**) and HemoCue®—QBC®-measured haemoglobin concentrations (**B**). * The pink boxes are the 95% confidence intervals around the mean difference and the limits of agreement

The mean HemoCue®-Coulter difference was − 0.15 g/dL (95% CI: − 0.22 to − 0.07 g/dL), for a SD of 1.80 g/dL and the standard error of 0.04 g/dL. The lower limit of agreement was − 3.68 g/dL (95% CI: − 3.80 to − 3.55 g/dL) and the upper limit of agreement was 3.39 g/dL (95% CI: 3.26 to 3.51 g/dL) (Fig. 2A).

### Kinshasa, DRC

The mean HemoCue®-QBC® difference was − 0.23 g/dL (95% CI: − 0.26 to − 0.20 g/dL), for a SD of 0.73 g/dL and the standard error of 0.06 g/dL. The lower and upper limits of agreement were − 1.66 g/dL (95% CI: − 1.71 to − 1.60 g/dL) and 1.20 g/dL (95% CI: 1.14 to 1.25 g/dL), respectively (Fig. 2B).

## Discussion

The study has shown that although the mean Hb differences between the paired methods were small, the limits of agreement between HemoCue®-measured and Coulter/QBC®-measured Hbs were wide, considerably wider than the somewhat stringent, predefined clinically acceptable limits of ± 0.5 g/dL, although there was a better agreement with the QBC® at KIMORU. Nevertheless, these results suggest suboptimal agreement between HemoCue® and Coulter/QBC®. The different measurement principles employed by HemoCue® and Coulter/QBC® likely played a role in this finding.

HemoCue® utilises a photometric measurement by analysing light absorption to determine the Hb concentration whilst the Coulter Counter relies on electrical impedance for blood cell counting and sizing and the QBC® measures the fluorescence intensity of the red blood cells in the buffy coat (higher intensity = higher Hb). Although the mean HemoCue®-Coulter Hb difference was very small and similar to the HemoCue®-QBC® mean difference, it is important to examine particularly the upper and lower limits of agreement because these values determine the degree of confidence clinicians will have before making important clinical decisions on patient management. These limits were broader in Mbale compared to Kinshasa, suggesting issues pertinent to the Coulter method e.g. suboptimal mixing of blood.

These findings contrast with those of Hinnouho et al. [25] (n = 1,487 paired measurements), who reported a higher mean Hb for the HemoCue® Hb-301™ (10.84 vs. 10.7 g/dL), for a mean difference of 0.87 g/dL but tighter limits of agreement of 0.6 to 1.14 g/dL compared to this study. They concluded poor agreement between HemoCue® Hb-301™ and Coulter Hb measurements. Similarly, Saghir et al*.* [28] study also reported a higher mean HemoCue®- and Coulter-measured Hb concentrations (14.6 vs. 13.9 g/dL, respectively) for a mean difference of 0.7 g/dL and broad limits of agreement of −2.1 to 2.1 g/dL. Their sample size of 455 paired measurements (vs. 1,880 in this study) probably resulted in a lower precision.

The findings align with the results reported by Adam et al. [29]. Comparing HemoCue® B-Haemoglobin vs. Coulter in a smaller study (n = 108 paired measurements), they reported broader limits of agreement of −2.36 to 5.04 g/dL and a higher mean difference of 1.34 g/dL whereas Bursey et al. [31] (n = 653 paired measurements) reported broadly similar limits of agreement to our study of − 2.00 to 2.00 g/dL but a much tighter mean difference of 0.008 g/dL; the mean Hbs were 9.1 (HemoCue®) and 9.2 g/dL (Coulter). Ranjitha et al. [30] (n = 200 paired measurements) demonstrated tight limits of agreement of − 0.37 to 0.25 g/dL and a low mean difference of − 0.01 g/dL between HemoCue® vs. Coulter counter [30]. They used predefined clinically acceptable limits of ± 1 g/dL, twice this study’s value, which may be clinically relevant in their context of patients undergoing neurosurgical procedures.

The findings are also consistent with studies conducted in various populations, namely, women with genetic Hb disorders in Cambodia [33], children in South Africa [34], blood donors in Ireland [35], and children and adults in Mexico [36]. These studies reported consistently lower mean HemoCue® Hbs compared to Coulter and limits of agreement that ranged from − 2.5 to 5.4 g/dL.

Several factors hinder the direct comparison of the study’s findings with previous research. One notable difference is the variation in the devices used. This study used the HemoCue® Hb-301™; others have used HemoCue® Hb-201™ or HemoCue® B-Haemoglobin™ and they employ distinct biochemical methods for Hb determination. The DxH500 Beckman Coulter Counter analysed the Mbale samples whilst previous studies used e.g. Beckman-Coulter LH 750, Sysmex KX21N, and Mindray BC-3000Plus. This may introduce small differences in Hb values even though the underlying principle remains the same. The study compared the QBC® method with HemoCue®, which, to the best of available evidence, has never been done previously.

Based on the study findings and those of others, it is evident that utilising HemoCue® as a screening tool for anaemia in children may yield inaccurate results and could significantly impact decision-making and patient care e.g., based on the data a child with a Coulter Counter-measured Hb concentration of 8.0 g/dL could have a HemoCue®-measured Hb concentration between 4.32 and 11.39 g/dL. Clinical signs have limited clinical value unless the patient is obviously very pale [20, 21]. The impact of an inaccurate HemoCue® measured Hb is likely to be greater at low Hb concentrations because this may lead to unnecessary referrals for further management of anaemia or unnecessary blood transfusions (with its attendant complications [37]) whilst overreading the true Hb concentration would result in a missed diagnosis of anaemia and no appropriate management of an anaemic patient. To avoid unnecessary blood transfusions, decisions on blood transfusion should be aided by the Consensus blood transfusion algorithm [38]. In this regard, it is important to define limits of agreement that best serve the clinical context in which the Hb is being measured. The study chose tight limits of agreement because the optimal management of anaemia in malaria-infected children was regarded as crucial.

One of the key strengths of this study is its large sample size, providing high precision in determining the agreement between the two methods. However, the study had several limitations. Firstly, the assumptions for conducting the Bland–Altman analysis, namely, the differences in Hb concentrations should be normally distributed and the confirmation of a non-linear relationship between the differences and the mean of measurements, were only approximately met. The study decided against log transforming the data due to the limitations of transformation, which do not facilitate inferences about the original data since it shares little with the original data [39] and makes interpreting results difficult. Moreover, the study did not measure Hb concentrations using the reference cyanmethaemoglobin method, making it impossible to determine which method had the greater accuracy.

## Conclusion

The study findings suggest that HemoCue® Hb-301™ cannot be used interchangeably with Coulter Counter and QBC®, given the wide limits of agreement. However, HemoCue® is still useful because it is low-tech and can be used in remote areas, but its results should be interpreted cautiously and must always take into account the clinical state of the patient. This is particularly important if a blood transfusion is being considered. More research is needed to identify the reasons for the observed discrepancies between the methods and explore ways to improve the agreement between these methods. In such comparisons, the true Hb values should be determined using the cyanmethaemoglobin method.

## Supplementary Information

Additional file1

## Data Availability

Data for this study and relevant supplementary data and documents (e.g. data dictionary, protocol, and participant information sheet) will be made available to applicants who provide a sound proposal to the Mahidol Oxford Tropical Medicine Research Unit Data Access Committee (datasharing@tropmedres.ac).

## Abbreviations

WHO: World Health Organization
DRC: Democratic Republic of Congo
KIMORU: Kinshasa Mahidol Oxford Tropical Medicine Research Unit
MRRH: Mbale Regional Referral Hospital
QBC: Quantitative Buffy Coat
HIV: Human immuno-deficiency virus
SMA: Severe malarial anaemia
CI: Confidence intervals
SD: Standard deviations
SE: Standard errors
