# Supplementary material for: Comparing HemoCue® and Quantitative Buffy Coat® and Coulter Counter-measured haemoglobin concentrations in African children with acute uncomplicated malaria: a Bland–Altman analysis
Source: Malar J. 2025 Mar 11;24:77. doi: 10.1186/s12936-025-05318-5 (PMC11895147; doi:10.1186/s12936-025-05318-5)
Supplement: Supplementary file 1 — Additional file1 [file 12936_2025_5318_MOESM1_ESM.docx]

**Additional file 1. Supplementary Figures for the Bland-Altman Analysis**

**Figure S1: Histograms of the distribution of baseline HemoCue®-measured haemoglobin concentrations in Mbale, Uganda (A) and Kinshasa, DRC (B).**

**A**


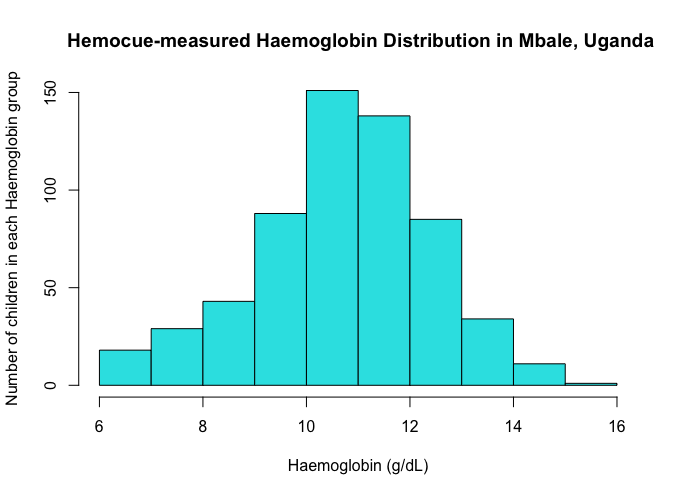


**B**


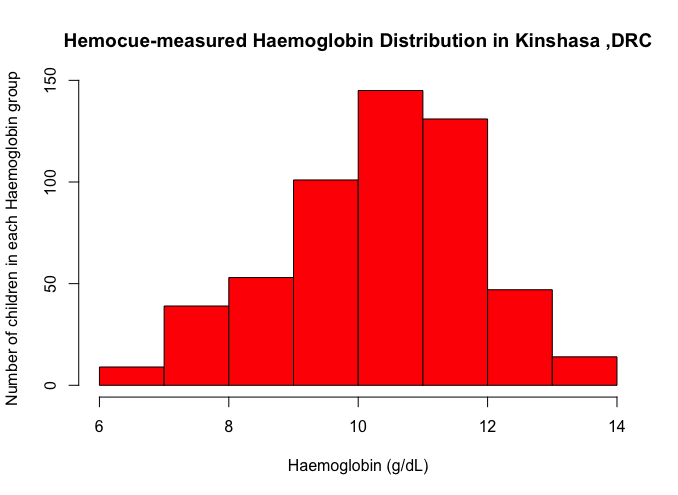


**Figure S2: Histograms of the distribution of baseline Coulter Counter and QBC**®**-measured haemoglobin concentrations in Mbale, Uganda (A) and Kinshasa, DRC (B)**

| **A**  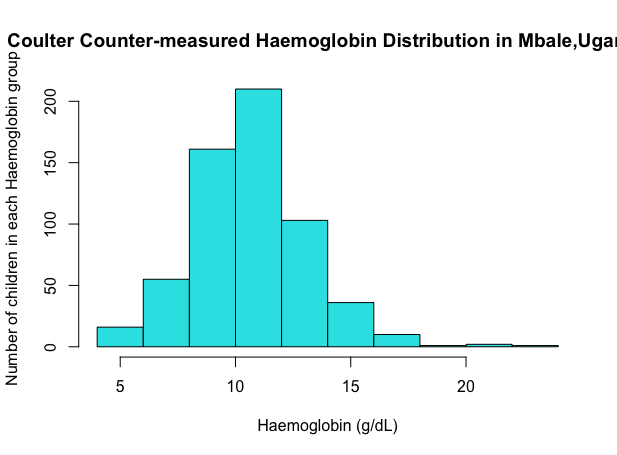 |
| --- |
| **B**  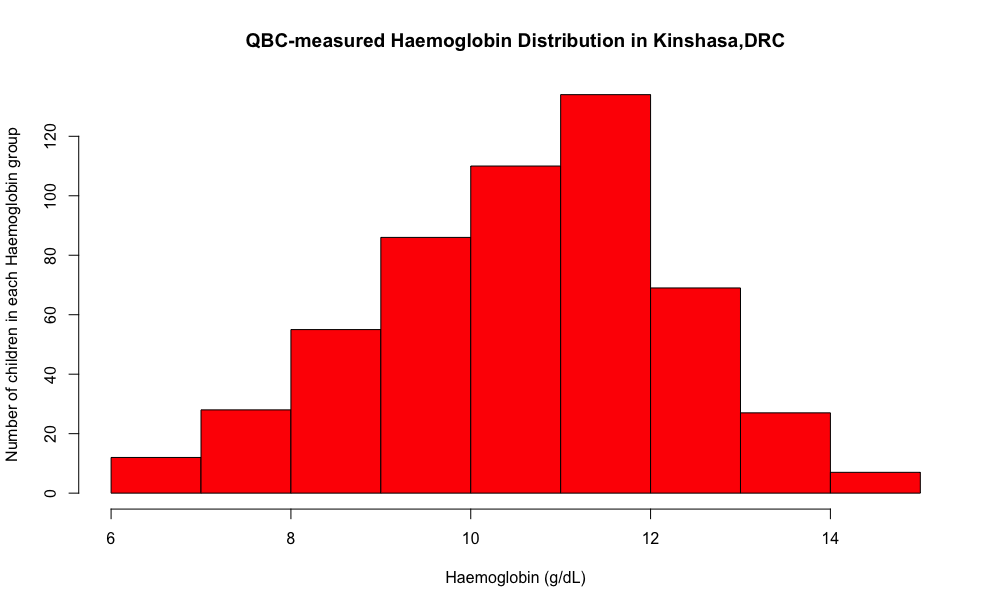 |

**Figure S3: Scatter plots of Counter-measured haemoglobin concentrations (A) and the QBC**®**-measured haemoglobin concentrations (B) vs. HemoCue®-measured haemoglobin concentrations.**

**A**


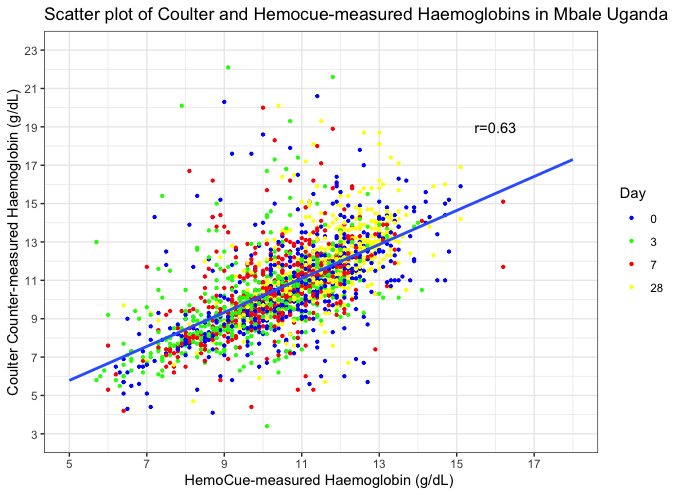


**B**


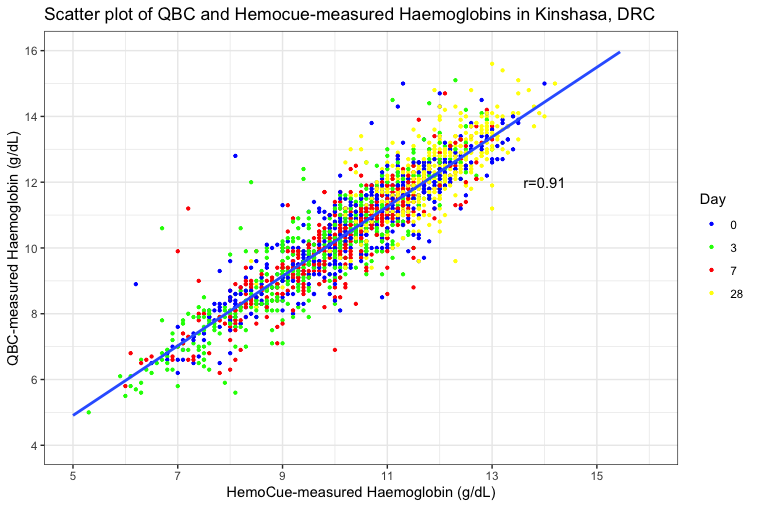


**Figure S4: Histograms of the distribution of the differences of HemoCue® - Coulter Counter (A) and HemoCue® - QBC**® **(B)-measured haemoglobin concentrations.**

| **A**  **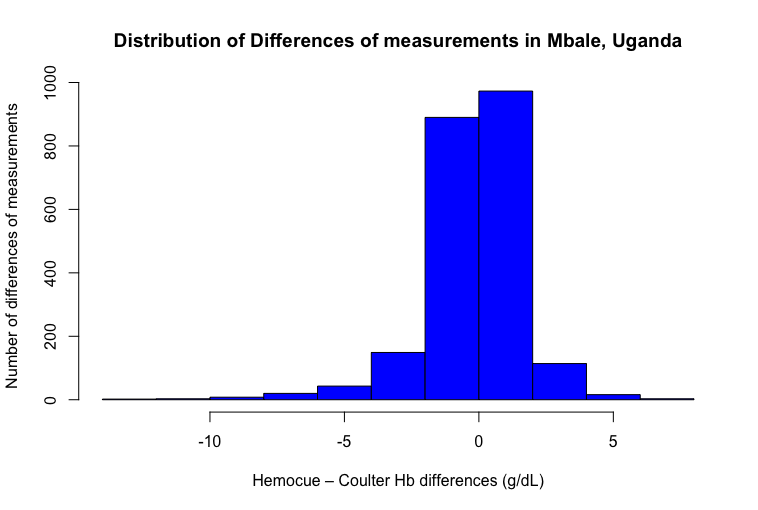** |
| --- |
| **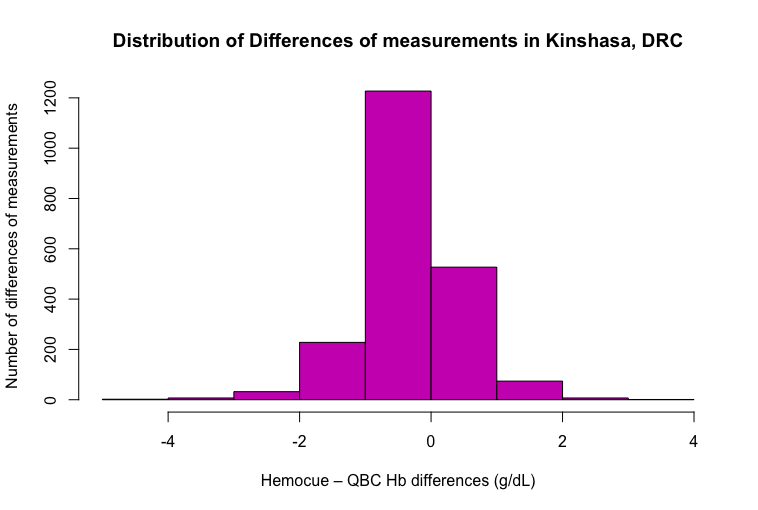**  ***B*** |

**Figure S5: Quantile-Quantile(Q-Q) *plots of* the differences of HemoCue® - Coulter Counter- (A) and HemoCue® - QBC**® **(B)-measured haemoglobin concentrations.**

| ***A***  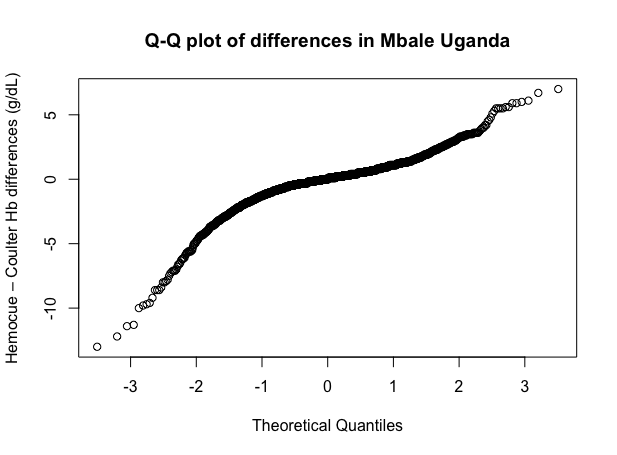 |
| --- |
| ***B***  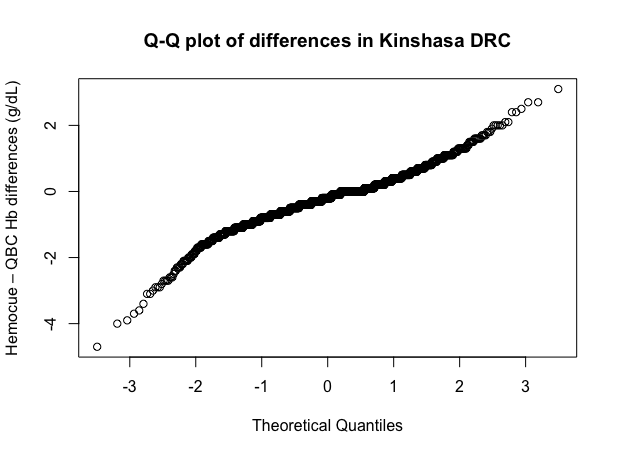 |

**Figure S6: Scatter plots of the differences of HemoCue® - Coulter Counter (A) and HemoCue® - QBC® (B)-measured haemoglobin concentrations, assuming the Coulter and QBC® methods are the reference methods.**

**A**


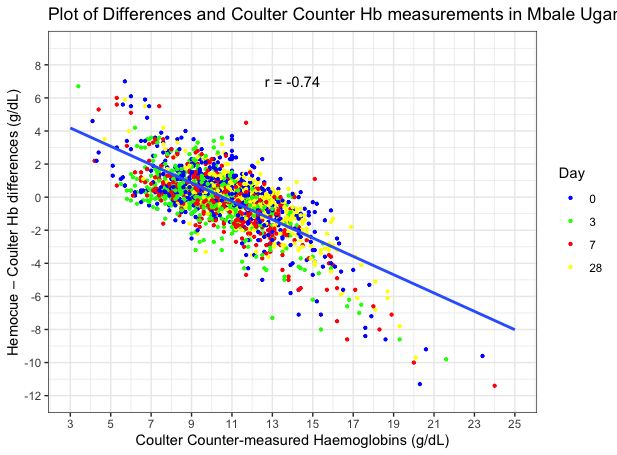


**B**


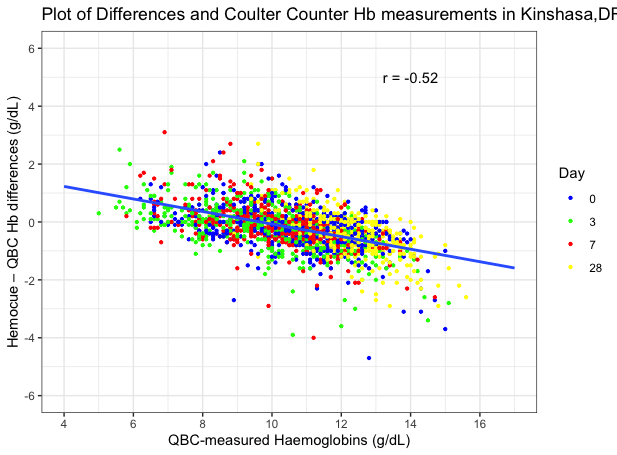


## Results of the analysis assuming coulter counter/QBC-measured haemoglobin concentrations are the reference values.

Assuming that the Coulter Counter/QBC is the “Gold standard”, measuring the true haemoglobin concentrations, plots of differences in the two measurements (HemoCue® minus QBC/Coulter Counter) against the Coulter Counter/QBC-measured values were made. The correlation coefficient between differences and Coulter Counter/QBC measurements for Kinshasa was -0.52, and Mbale was -0.74, respectively. These were high and moderate negative correlations [40]; hence, a significant negative relationship existed between the differences and the Coulter Counter-measured values, as shown in Figure 6. This signifies the presence of systematic and proportional errors even if the Coulter Counter/QBC was measuring the true values.

On the other hand, the Bland-Altman analysis gave the same mean difference and limits of agreement as when the means of the two measurement values were used to estimate the true values. Only the correlation coefficients changed, as shown in Figure 7 below.

**Figure S7: Bland-Altman plots of Coulter counter (A) or QBC®-measured (B)and HemoCue®*-measured h*aemoglobin concentrations, assuming the Coulter counter or *the* QBC**® **values are the reference values.**

**A**

| **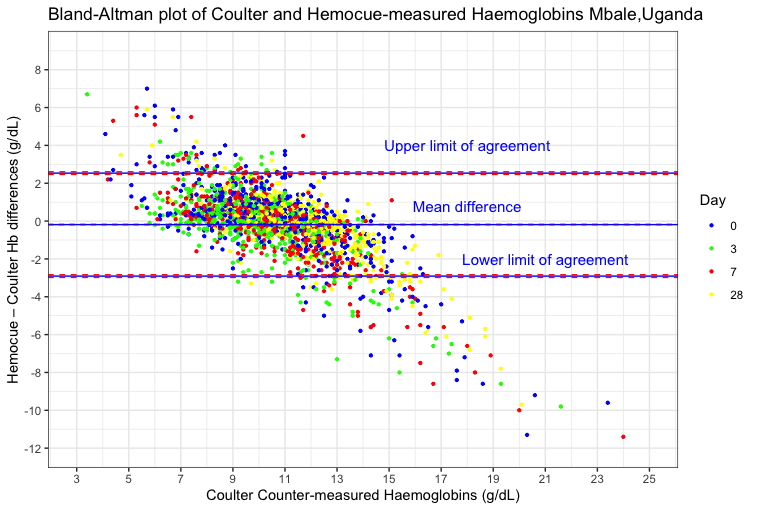** |
| --- |
| **B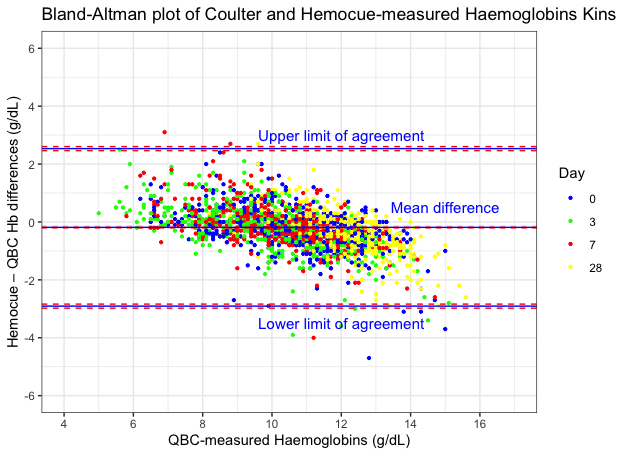** |
